# Reciprocal effects of treatment-induced increases in exercise and improved eating, and their psychosocial correlates, in obese adults seeking weight loss: a field-based trial

**DOI:** 10.1186/1479-5868-10-133

**Published:** 2013-12-05

**Authors:** James J Annesi, Kandice J Porter

**Affiliations:** 1 YMCA of Metropolitan Atlanta, 100 Edgewood Avenue, NE, Suite 1100, Atlanta, GA 30303, USA; 2 Department of Health Promotion and Physical Education, Kennesaw State University, 1000 Chastain Road, Kennesaw, GA 30144, USA

**Keywords:** Exercise, Nutrition, Cognitive-behavioral, Obesity, Reciprocal effects

## Abstract

**Background:**

A better understanding of interrelations of exercise and improved eating, and their psychosocial correlates of self-efficacy, mood, and self-regulation, may be useful for the architecture of improved weight loss treatments. Theory-based research within field settings, with samples possessing high probabilities of health risks, might enable rapid application of useful findings.

**Methods:**

Adult volunteers with severe obesity (body mass index [BMI] 35–50 kg/m^2^; age = 43.0 ± 9.5 y; 83% female) were randomly assigned to six monthly cognitive-behavioral exercise support sessions paired with either group-based nutrition education (n = 145) or cognitive behavioral methods applied to improved eating (n = 149). After specification of mediation models using a bias-corrected bootstrapping procedure, a series of reciprocal effects analyses assessed: a) the reciprocal effects of changes in exercise and fruit and vegetable intake, resulting from the treatments, b) the reciprocal effects of changes in the three psychosocial variables tested (i.e. self-efficacy, mood, and self-regulation) and fruit and vegetable change, resulting from change in exercise volume, and c) the reciprocal effects of changes in the three psychosocial variables and exercise change, resulting from change in fruit and vegetable intake.

**Results:**

Mediation analyses suggested a reciprocal effect between changes in exercise volume and fruit and vegetable intake. After inclusion of psychosocial variables, also found were reciprocal effects between change in fruit and vegetable intake and change in mood, self-efficacy for controlled eating, and self-regulation for eating; and change in exercise volume and change in mood and exercise-related self-regulation.

**Conclusion:**

Findings had implications for behavioral weight-loss theory and treatment. Specifically, results suggested that treatments should focus upon, and leverage, the transfer effects from each of the primary weight-loss behaviors (exercise and healthy eating) to the other. Findings on psychosocial correlates of these behavioral processes may also have practical applications.

## Background

Obesity is an extreme problem that continues to elevate health risks [1]. Although the universal prescription for reducing overweight remains replacing high-fat and high-calorie diets with more healthy eating and increasing exercise outputs, these behaviors have been *highly* resistant to change. Typically, individuals are provided information on appropriate eating practices and the need to exercise, which lacks a strong theoretical basis for behavioral change. Any improvements have largely been transient [2,3]. Although the use of cognitive-behavioral methods have had somewhat better behavior-change outcomes [4,5], it is still rare for weight loss of even 5 to 10% of one’s original body weight to be maintained beyond the short term [3]. After the unexpected failure of their recent state-of-the-art cognitive-behavioral treatment that intensively focused on sustaining weight loss through self-monitoring, Cooper et al. questioned whether there is sufficient evidence to suggest that *any* behavioral approach will be effective, and suggested a “… shift away from work on treatment and instead focus on prevention” [6] p. 712. Consistent with this stark viewpoint, invasive and expensive surgical methods (with considerable health risks themselves, and questionable long-term effects) are becoming the treatment of choice for obesity [7,8].

In their comprehensive review, Mann et al. [3] also acknowledged the overall failure of behavioral weight-loss treatments, but suggested that exercise may have promise in areas yet to be adequately explored. Exercise is the most robust predictor of maintained weight loss [9,10]. However, because obese and sedentary individuals can complete only minimal volumes [11], it is likely that the benefits of exercise for weight management transcend its associated energy expenditure. Unfortunately, adherence to exercise programs is also typically low [12]. Less that 5% of American [13,14] and Canadian [15] adults complete even the minimum recommended amount, even though that is equivalent to only several walks per week [16].

Because of the apparently poor understanding of how to best induce meaningful change in exercise and eating behaviors, researchers have been encouraged to turn to established behavior-change theory to adjust their intervention methods [17]. For example, social cognitive theory [18,19] suggests both direct and reciprocal relationships between exercise, healthy eating, changes in psychological variables, and weight loss that require greater consideration. Almost two decades ago, Friedman and Brownell [20] recommended that research progress to a “third generation of studies” that focuses on the behavioral treatment of obesity through analyses of causal mechanisms and interactions between psychosocial variables and weight-loss behaviors. Subsequently, Baker and Brownell [21] posited that a relationship between exercise and improved eating and weight loss was mediated by improved mood, body image, self-efficacy (feelings of ability to complete a task), self-esteem, and coping. Annesi and Marti [22] extended those propositions through path analysis, and suggested that changes in self-efficacy, mood, and use of self-regulatory skills are associated with both exercise and improved eating, and there might be a carry-over from exercise-induced psychosocial changes to changes in eating and weight loss (e.g. use of self-regulatory skills for exercise generalizes to self-regulation for eating). Variants of these propositions were suggested in research from Finland [23] and Portugal [24,25]. Although this provided a partial explanation of relationships between exercise, improved eating, and weight management (beyond caloric expenditure), synergistic changes of the behaviors via effects on their proposed psychosocial correlates were unclear.

The interrelationships of the two key behaviors for the successful treatment of obesity, eating and exercise, are important, but poorly understood. For example, a cognitive-behavioral intervention might be effective partially because it improves eating behaviors (e.g. daily intake of fruits and vegetables), which in turn may help to induce an increased volume of weekly exercise. Concurrently, increased exercise might lead to better eating behaviors. Determination of the psychological foundation of such relationships might substantially inform both weight-loss theory and treatment development. For example, increased exercise volume may induce improved eating partially because it improves mood; while, concurrently, better eating behaviors might have mood-improving properties. These propositions are consistent with both social cognitive theory and self-efficacy theory [18,26]. Self-efficacy and self-regulation (internal means to control or alter one’s behaviors), also key constructs of the aforementioned theories, might be additional psychosocial variables appropriate for similar consideration. A greater understanding of such relationships could be facilitated through reciprocal effects analysis, which is an extension of traditional mediation analysis [27,28] initially demonstrated in a recent article in International Journal of Behavioral Nutrition and Physical Activity (that used the methodology for assessing behavioral weight-loss processes) [29]. More specifically, such analyses could help to establish whether changes in psychosocial variables such as self-efficacy, mood, and self-regulation have reciprocal relationships with exercise and eating changes (e.g. improved self-efficacy could be consistent with more exercise, while more exercise could yield more self-efficacy) [30].

In the present research context of two behavioral treatments for weight loss that are focused on supporting exercise and healthy eating (one emphasizing self-regulation methods for controlled eating, and one emphasizing nutrition education), this investigation was conducted. A field setting incorporating severely obese adults was selected because findings may be especially applicable to this group that experiences considerable health risks. In regard to treatment-related results, the following was expected: a) volume of exercise, fruit and vegetable intake, mood, and exercise- and eating-related self-efficacy and self-regulation would significantly improve over the 26-week study, and b) self-regulation for eating and fruit and vegetable intake would be significantly greater in the group emphasizing self-regulation methods for eating. In regard to relationships within variables, the following reciprocal effects were hypothesized: a) treatment-associated change in fruit and vegetable intake would both mediate and be mediated by change in exercise volume; b) changes in mood, self-efficacy for controlled eating, and self-regulation for eating would both mediate and be mediated by change in fruit and vegetable intake, with change in exercise as the predictor variable, and c) changes in mood, exercise self-efficacy, and self-regulation for exercise would both mediate and be mediated by change in exercise volume, with fruit and vegetable intake as the predictor.

It was hoped that findings would lead to a better understanding of the relationship between increased exercise and improved eating, and how theory-based psychosocial variables that affect this relationship may, ultimately, be targeted to improve weight-loss treatments.

## Methods

### Participants

Individuals responded to print advertisements soliciting volunteers for research on the use of exercise and nutrition instruction for weight loss to be completed in YMCA centers in the southeastern U.S. Inclusion criteria were: a) minimum age of 21 y, b) BMI of 35–50 kg/m^2^, and c) no regular exercise (less than a self-reported mean of 30 min/week). Exclusion criteria were: a) current enrollment in a commercial or medical weight-loss program, b) soon-planned or current pregnancy, and c) taking medications prescribed for weight loss or a psychological condition that might affect survey responses (e.g. anxiety disorder, depression). A signed statement of adequate health to participate was required from a physician. Approval was received from the institutional review board of Kennesaw State University, and written informed consent was appropriately obtained from all participants.

**Figure 1:**
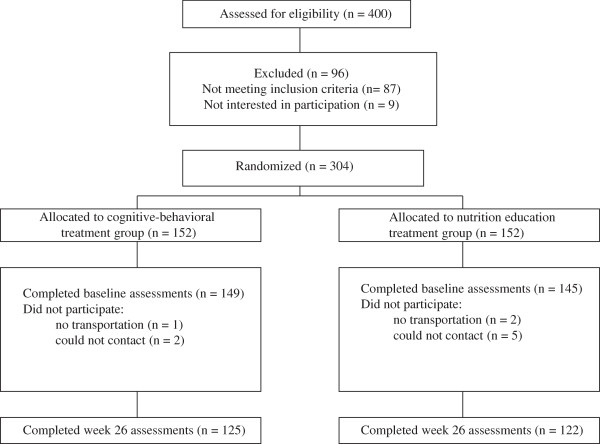
Flow of participants through the investigation.

Figure 1 outlines the flow of participants through the recruitment and study processes. There was no significant difference in proportion of women (overall 83%), age (overall 43.0 ± 9.5 y), BMI (overall M = 40.5 ± 4.1 kg/m^2^), and racial make-up (overall 48% White, 48% African American, and 4% of other racial/ethnic groups) between participants assigned to a treatment of cognitive-behavioral exercise support plus nutrition education (nutrition education group; n = 145) and the same cognitive-behavioral exercise support plus cognitive-behavioral nutrition methods (cognitive-behavioral nutrition group; n = 149) through simple random sampling. Nearly all participants were middle-class.

### Measures

#### *Exercise behavior*

Volume of exercise was measured by the Godin-Shephard Leisure-Time Physical Activity Questionnaire [31]. It incorporates estimates of metabolic equivalent of tasks (METs), or the physiological energy cost based on physical activity intensity. Respondents enter weekly frequencies of strenuous (“heart beats rapidly”, e.g. running), moderate (“not exhausting”, e.g. fast walking), and light (“minimal effort”, e.g. easy walking) exercise for “more than 15 minutes” per session. The responses are multiplied by 9, 5, and 3 METs, respectively, and then summed. Reported test-retest reliability over 2 weeks was .74 [32]. Construct validity was previously indicated by significant correlations with accelerometer and maximum volume of oxygen uptake assessments [33,34].

#### *Eating behavior*

Intake of number of fruits and vegetable servings “in a typical day” (“looking back over the last month”) was based on the U.S. Food Guide Pyramid’s descriptions of foods and their corresponding portion sizes, and how to count “mixed foods” (e.g. cereal with fruit; salads) [35]. Responses from the two items (one each for fruits and vegetables) were summed. Reported test-retest reliability over 2 weeks averaged .82, and concurrent validity was indicated through significant correlations of the present measure with lengthier food frequency questionnaires [35]. In pilot research, predictive validity was supported in severely obese adults through a significant inverse relationship (r = -.42, p < .001) between change in this measure and weight change. Research suggests that fruit and vegetable consumption, alone, is an accurate predictor of quality of the diet and overall caloric consumption [36,37], and in contexts such as the present research, single-item scales may not possess a disadvantage [38].

#### *Self-efficacy*

Self-efficacy for controlled eating was measured by the Weight Efficacy Lifestyle Scale [39]. It incorporates items from five factors (negative emotions, availability, physical discomfort, positive activities, and social pressure) (e.g. “I can resist eating even when others are pressuring me to eat”), that are summed for a total score. Responses to the scale’s 20 items range from 0 (not confident) to 9 (very confident). Internal consistency was reported to range from α = .70-.90 [39]. Internal consistency for the present sample was α = .82.

Exercise self-efficacy was measured by the Exercise Self-Efficacy Scale [40]. Five items begin with the stem, “I am confident I can participate in regular exercise when …” (e.g. “I feel I don’t have the time)”, with responses ranging from 1 (not at all confident) to 11 (very confident). Reported internal consistency ranged from α = .76-.82, and test-retest reliability over 2 weeks was .90 [41]. Internal consistency for the present sample was α = .84.

#### *Mood*

Mood was assessed by the Profile of Mood States Short Form’s measure of Total Mood Disturbance [42]. It is an aggregate of six subscales (Depression, Tension, Fatigue, Vigor, Confusion, and Anger) incorporating a total of 30, one- to three-word items (e.g. “sad”, “worn out”, “tense”) that are rated from 0 (not at all) to 4 (extremely) based on, “how you have been feeling during the past week including today”. Internal consistency ranged from α = .84-.95, and test-retest reliability at 3 weeks averaged .69 [42]. Internal consistency for the present sample was α = .79-.91.

#### *Self-regulation*

For the measurement of self-regulatory skill usage for exercise and self-regulatory skill usage for controlled eating, a scale by Saelens et al. [43], where items are to be based on the content of the intervention presently being used, was adapted. Examples of the 10-item scales are, “I set physical activity goals”, and “I purposefully address my barriers to eating appropriately”, respectively. Responses range from 1 (never) to 5 (often). Reported internal consistency was α = .79 and .81, respectively; and test-retest reliability over 2 weeks was .78 and .74, respectively [22]. Internal consistency for the present sample was α = .76 and α = .79, respectively.

### Procedure

Each participant reported to his/her assigned YMCA center, received a group orientation to the study’s processes, and was provided access to the facility for the duration of the study. The cognitive-behavioral exercise support component was identical for each of the two groups. It consisted of a computer-supported protocol of six, 45- to 60-min meetings (approximately monthly) with a trained wellness specialist over 26 weeks [12]. These sessions included an orientation to exercise areas and apparatus, but most time was spent in individual consultation in an office. Long-term goals were identified and broken down into process-oriented short-term goals. Goal progress was tracked graphically on the computer at each meeting. Self-regulatory skills instructions addressed cognitive restructuring, stimulus control, behavioral contracting, and relapse prevention. Exercise modalities (e.g. treadmill; walking on an indoor track) were based on each participant’s preference. Standard exercise recommendations (i.e. 150 minutes/week of moderate cardiovascular activity [16]) were described; however, the benefit from *any* volume of exercise was also indicated.

The nutrition components differed by group, with one emphasizing education in healthy eating practices, and one emphasizing the use of self-regulation methods to control inappropriate eating. Each had six, 1-hour sessions administered by a certified wellness specialist in group format of 10–15 participants over 12–14 weeks. In the nutrition education group, the standardized protocol [44] included: a) understanding macronutrients and calories, b) healthy recipes, c) menu planning, d) low-fat snacking, and e) stocking a healthy kitchen. In the cognitive-behavioral nutrition group, the protocol included: a) establishing caloric goals based on weight, b) logging foods and associated calories, c) cognitive restructuring, d) relapse prevention training, and e) cues to overeating. In both groups, increasing exercise and fruit and vegetable intake was emphasized during each session.

Wellness specialists that administered the treatments had YMCA and other national health and fitness certifications, and were blind to the purposes the study. Approximately 15% of treatment sessions were monitored for fidelity by study staff members. Deviations from the assigned protocols rarely occurred. However, when indicated, corrective measures were immediately taken by YMCA supervisors in cooperation with study administrators. Assessments were administered in a private area at baseline and week 26.

### Data analyses

An intention-to-treat format was incorporated where data from all participants initiating treatment were included in the analyses. The 16% of missing measure scores (all missing at week 26) were imputed using the expectation-maximization algorithm [45,46]. Statistical significance was set at α = .05 (two-tailed), throughout. Effect sizes are expressed as either Cohen’s d or partial eta-square (η^2^_p_) where .20, .50, and .80; and .01, .06, and .14 denote small, moderate, and large effects, respectively. For the planned multiple regression analysis, to detect a small-to-moderate effect (f^2^ = .08) at the statistical power of .90, α = .05, a minimum of 180 participants was required [47]. Oversampling was conducted to secure the statistical power.

Scores of all measures were approximately normally distributed. Change scores in measures of exercise volume, fruit and vegetable intake, mood, and exercise- and eating-related self-regulation and self-efficacy were calculated as differences between scores at baseline and scores at week 26. As suggested for the present research context [48], change scores were unadjusted for their baseline value. Mixed model repeated measure ANOVAs (time × treatment type) were incorporated to simultaneously assess whether the changes in each variable were significant over 26 weeks, and whether those changes differed across the two treatment types.

Using aggregated data, intercorrelations of scores were computed, and multiple regression models were fitted to separately assess the independence of changes in the measures of self-regulation, self-efficacy, and mood in the prediction of changes in exercise volume and fruit and vegetable intake. Collinearity was tested, and both the variance inflation factors (1.27-1.74) and tolerances (.43-.79) were within accepted limits for regression analyses. Inspection of residual scatterplots indicated homogeneity of variance and linearity in the data.

**Figure 2:**
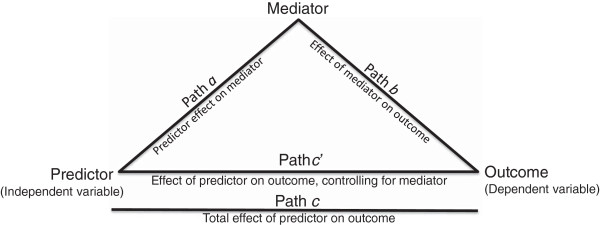
Guide for interpretation of mediation analyses.

Mediation models (see Figure 2) were specified using a bias-corrected bootstrapping procedure incorporating 10,000 re-samples [28]. Within this procedure, R^2^ is used to assess significance of overall mediation, and if the relationship of the predictor and outcome variable (path c) is no longer significant after entry of the mediator (path c′), then *complete mediation* is identified. Utilizing this mediation analysis method [28], a series of reciprocal effects analyses, based on recent related research [29], were computed that assessed: a) the reciprocal effects of changes in exercise and fruit and vegetable intake, resulting from the two treatment types, b) the reciprocal effects of changes in the three psychosocial variables of interest (i.e. self-efficacy, mood, and self-regulation) and fruit and vegetable change, resulting from change in exercise volume, and c) the reciprocal effects of changes in the three psychosocial variables and exercise change, resulting from change in fruit and vegetable intake. Considering the rationale presented by Marsh and Craven [49], a reciprocal effect was considered to be present when mediation is significant in each of two complementary models having a consistent predictor -- one model where a variable is specified as an outcome, and the other where that same variable is specified as a mediator [29]. For example, in the initial reciprocal effects analysis, treatment type was the predictor in both mediation models where, in the first equation of that analysis, change in exercise volume was the outcome variable and change in fruit and vegetable intake was the mediator. In the second and complementary equation of that analysis, fruit and vegetable intake change was the outcome variable and change in exercise was the mediator. The same procedure was then used to assess the presence of six additional reciprocal relationships that incorporated psychosocial factors that emerged from both social cognitive theory [18,19] and previous research [22-25]. In the initial three of these reciprocal effects analyses, change in exercise volume was the predictor, fruit and vegetable intake change was the outcome measure, and either change in the measure of self-efficacy, mood, or self-regulation was the mediator in its initial equation, and then was the outcome variable in its complementary equation. In the final three reciprocal effects analyses, change in fruit and vegetable intake was the predictor and volume of exercise change was the outcome variable in its initial equation, with the same pattern of entry of the psychosocial variables as in the previous three reciprocal effects analyses.

## Results

Descriptive statistics of study measures at baseline and week 26, mean change scores, and their effect sizes are given in Table 1. There were no significant differences between the treatment types at baseline in any study variable (ps > .15). Significant effects for time were found over 26 weeks in all measures (ps < .001), indicating overall significant improvements. There was a time × treatment type interaction for volume of exercise (F(1, 292) = 4.11, p = .04, η^2^_p_ = .01), fruit and vegetable intake (F(1, 292) = 10.24, *p* < .01, η^2^_p_ = .03), and self-regulation for eating (F(1, 292) = 7.72, *p* < .01, η^2^_p_ = .03), indicating greater improvements in those variables associated with the cognitive-behavioral nutrition group.

**Table 1:** Changes in study measures over 26 weeks

	**Baseline**		**Week 26**				
	**M**	**SD**	**M**	**SD**	**M**_**change**_	**SD**	**d**
Exercise volume (METs)							
Nutrition education group	7.67	6.58	20.20	16.82	12.53	15.84	1.90
Cognitive-behavioral nutrition group	6.91	6.91	23.57	18.42	16.66	18.87	2.41
Overall sample	7.29	6.75	21.92	17.70	14.63	17.54	2.17
Fruit and vegetable intake							
Nutrition education group	4.48	2.05	4.91	2.19	0.44	1.37	.21
Cognitive-behavioral nutrition group	4.18	1.78	5.19	1.96	1.01	1.68	.57
Overall sample	4.33	1.92	5.05	2.08	0.73	1.55	.38
Self-efficacy for controlled eating							
Nutrition education group	99.41	34.61	114.93	34.00	15.52	27.98	.45
Cognitive-behavioral nutrition group	96.94	36.01	114.97	38.81	18.32	30.70	.51
Overall sample	98.01	35.29	114.95	36.46	16.94	29.37	.48
Self-regulation for eating							
Nutrition education group	21.28	5.82	24.82	6.92	3.54	5.52	.61
Cognitive-behavioral nutrition group	21.60	5.59	26.94	7.10	5.34	5.62	.96
Overall sample	21.44	5.70	25.89	7.08	4.45	29.37	.78
Mood							
Nutrition education group	22.58	17.29	14.04	18.96	-8.53	14.19	.49
Cognitive-behavioral nutrition group	21.99	17.00	10.68	17.21	-11.31	15.38	.67
Overall sample	22.28	17.12	12.34	18.14	-9.94	14.84	.58
Exercise self-efficacy							
Nutrition education group	29.95	12.01	33.21	11.53	3.26	10.05	.27
Cognitive-behavioral nutrition group	30.26	11.59	32.44	11.81	2.18	9.96	.19
Overall sample	30.11	11.78	32.82	11.66	2.71	10.00	.23
Self-regulation for exercise							
Nutrition education group	19.96	5.67	25.86	7.79	5.90	7.78	1.04
Cognitive-behavioral nutrition group	20.79	4.86	27.38	7.22	6.58	7.50	1.49
Overall sample	20.38	5.28	26.63	7.53	6.25	7.63	1.18

Note. Nutrition education group n = 145. Cognitive-behavioral nutrition group n = 149. Overall sample N = 294. d denotes Cohen’s effect size for within-group change: M_week 26_ - M_baseline_/SD_baseline_.

Change scores on the variables demonstrated low to moderate intercorrelations (Table 2). An exception to this was the moderate-strong correlation between the two measures of self-regulatory skills use (.69), which were still kept separate based on theory (i.e. self-regulation being domain-specific [50]). In multiple regression analyses, simultaneous entry of changes in the measures of exercise self-efficacy, mood, and self-regulation for exercise accounted for a significant portion of the variance in change in exercise volume (R^2^ = .43, F(3, 290) = 71.61, p < .001). Each psychosocial variable independently explained a significant portion of the variance in change in exercise volume, while controlling for the other variables (β(SE) = .11(.08), p = .03; -.27(.06), p < .001; and .42(.13), p < .001, respectively). Simultaneous entry of changes in the measures of self-efficacy for controlled eating, mood, and self-regulation for eating accounted for a significant portion of the variance in change in fruit and vegetable intake (R^2^ = .21, F(3, 290) = 26.17, p < .001). Again, each independently explained a significant portion of the variance in eating change, while controlling for the other variables (β(SE) = .26(.003), p < .001; -.16(.01), p = .02; and .13(.02), p = .05, respectively).

**Table 2:** Intercorrelations among study measures (N = 294)

	**1**	**2**	**3**	**4**	**5**	**6**	**7**
1. ΔExercise volume							
2. ΔFruit and vegetable intake	.31^†^						
3. ΔSelf-efficacy for controlled eating	.43^†^	.42^†^					
4. ΔSelf-regulation for eating	.52^†^	.35^†^	.53^†^				
5. ΔMood	-.52^†^	-.37^†^	-.55^†^	-.54^†^			
6. ΔExercise self-efficacy	.32^†^	.05	.47^†^	.32^†^	-.26^†^		
7. ΔSelf-regulation for exercise	.60^†^	.40^†^	.59^†^	.69^†^	-.55^†^	.36^†^	

Note. The Delta symbol (Δ) denotes change from baseline to week 26.

^†^p < .01.

Table 3 shows results from the reciprocal effects analyses. Figure 2 provides a guide to enhance interpretation. As described previously, if each of the two paired models demonstrates significant mediation, then reciprocal effects was identified. In reciprocal effects analysis 1, change in fruit and vegetable intake significantly mediated the relationship between treatment type and change in exercise volume (complete mediation), and change in exercise significantly mediated the relationship between treatment type and change in fruit and vegetable intake. Thus, results were consistent with the presence of a reciprocal effect between change in exercise volume and change in fruit and vegetable intake (and resulting from treatment type). In reciprocal effects analysis 2–4, the relationship between changes in volume of exercise and fruit and vegetable intake were significantly mediated, in separate models, by changes in self-efficacy for controlled eating, mood, and self-regulation for eating. Also, change in fruit and vegetable intake was a significant mediator of the relationship between exercise volume change, and change in the corresponding psychosocial variable. Thus, for reciprocal effects analysis 2, 3, and 4, results were consistent with the presence of a reciprocal effect between change in fruit and vegetable intake and change in each of the three psychosocial variables tested (and resulting from change in volume of exercise).

**Table 3:** **Results from mediation and reciprocal effects analyses (N** ***=*** **294)**

**Predictor**	**Mediator**	**Outcome**	**Path a**	**Path b**	**Path c**	**Path c’**	**Indirect effect**	**R**^**2**^
			**Coef (SE)**	**Coef (SE)**	**Coef (SE)**	**Coef (SE)**	**Coef (SE)**	
							**95% CI**	
Analysis 1^◊^								
Treatment	ΔFruit & Vegtable	ΔExercise	.57(.18)^†^	3.33(.64)^†^	4.23(2.04)*	2.23(1.98)	1.89(.70)	.10^†^
							.73, 3.53	
Treatment	ΔExercise	ΔFruit & Vegetable	4.13(2.04)*	.03(.01)^†^	.57(.18)^†^	.46(.17)^†^	.10(.06)	.12^†^
							.01, .24	
Analysis 2^◊^								
ΔExercise	ΔSelf-efficacy-Eat	ΔFruit & Vegetable	.71(.09)^†^	.02(.00)^†^	.03(.01)^†^	.01(.01)^†^	.01(.00)	.19^†^
							.01, .02	
ΔExercise	ΔFruit & Vegetable	ΔSelf-efficacy-Eat	.03(.01)^†^	5.98(1.00)^†^	.71(.09)^†^	.55(.09)^†^	.16(.05)	.27^†^
							.08, .26	
Analysis 3^◊^								
ΔExercise	ΔMood	ΔFruit & Vegetable	-.44(.04)^†^	-.03(.01)^†^	.03(.01)^†^	.01(.01)*	.01(.00)	.16^†^
							.01, .02	
ΔExercise	ΔFruit & Vegetable	ΔMood	.03(.01)^†^	-2.25(.49)^†^	-.44(.04)^†^	-.38(.04)^†^	-.06(.02)	.32^†^
							-.09, -.03	
Analysis 4^◊^								
ΔExercise	ΔSelf-regulate-Eat	ΔFruit & Vegetable	.17(.02)^†^	.07(.02)^†^	.03(.01)^†^	.02(.01)^†^	.01(.00)	.15^†^
							.01, .02	
ΔExercise	ΔFruit & Vegetable	ΔSelf-regulate-Eat	.03(.01)^†^	.78(.19)^†^	.16(.02)^†^	.15(.02)^†^	.02(.01)	.31^†^
							.01, .04	
Analysis 5								
ΔFruit & Veg	ΔExercise self-efficacy	ΔExercise	.34(.38)^†^	.54(.09)^†^	3.46(.63)^†^	3.28(.60)^†^	.18(.29)	.18^†^
							-.34, .82	
ΔFruit & Veg	ΔExercise	ΔExer self-efficacy	3.46(.63)^†^	.20(.03)^†^	.34(.38)	-.34(.38)	.68(.20)	.11^†^
							.36, 1.17	
Analysis 6^◊^								
ΔFruit & Veg	ΔMood	ΔExercise	-3.58(.52)^†^	-.56(.06)^†^	3.46(.63)^†^	1.46(.60)*	2.01(.38)	.29^†^
							1.37, 2.84	
ΔFruit & Veg	ΔExercise	ΔMood	3.46(.63)^†^	-.38(.04)^†^	-3.58(.52)^†^	-2.25(.49)^†^	-1.32(.34)	.32^†^
							-2.04, -.72	
Analysis 7^◊^								
ΔFruit & Veg	ΔSelf-regulate-Ex	ΔExercise	1.96(.26)^†^	1.31(.12)^†^	3.46(.63)^†^	.89(.58)	2.57(.59)	.37^†^
							1.60, 3.62	
ΔFruit & Veg	ΔExercise	ΔSelf-regulate-Ex	3.46(.63)^†^	.23(.02)^†^	1.96(.23)^†^	1.16(.23)^†^	.80(.17)	.41^†^
							.46, 1.15	

Note. The Delta symbol (Δ) denotes change from baseline to week 26. Path a = predictor → mediator;

Path b = mediator → outcome; Path c = predictor → outcome; Path c’ = predictor → outcome (controlling for the mediator).

*p < .05, ^†^p < .01.

^◊^denotes a reciprocal effect between the mediator and outcome variables (resulting from the predictor).

In reciprocal effects analysis 5, exercise self-efficacy was not a significant mediator of the relationship between changes in fruit and vegetable intake and exercise volume. Thus, a reciprocal effect was not detected. In reciprocal effects analysis 6 and 7, the relationship between changes in fruit and vegetable intake and volume of exercise were significantly mediated, in separate models, by changes in mood and self-regulation for exercise (the latter demonstrating complete mediation). Also, change in exercise was a significant mediator of the relationship between fruit and vegetable intake change and change in each corresponding psychosocial variable. Thus, for reciprocal effects analysis 6 and 7, results were consistent with the presence of a reciprocal effect between change in volume of exercise and change in mood and self-regulation (and resulting from change in fruit and vegetable intake).

## Discussion

This study was conducted to provide a better understanding of how psychosocial variables such as self-regulation, mood, and self-efficacy may affect relationships between increased exercise and improved eating in order that that weight-loss treatments may, ultimately, be improved. Findings suggested that change in exercise volume and change in fruit and vegetable consumption (the present measure of quality of eating) have a reciprocal relationship where each behavior reinforces the other. Also found were reciprocal effects between change in fruit and vegetable intake and changes in mood, self-efficacy for controlled eating, and self-regulation for eating; and change in exercise volume and changes in mood and exercise-related self-regulation.

The hypothesis that volume of exercise, fruit and vegetable intake, mood, and exercise- and eating-related self-efficacy and self-regulation would significantly improve was supported. The finding that the cognitive-behavioral nutrition treatment was associated with significantly greater improvements in both self-regulation for eating and fruit and vegetable intake than the educational approach was expected due to the corresponding content of that treatment protocol. The independence of changes in self-efficacy, mood, and self-regulation for the prediction of both increased volumes of exercise and greater intake of fruits and vegetables was consistent with both theory [18] and recent research [22-25].

The anticipated reciprocal relationship between treatment-induced changes in exercise volume and fruit and vegetable intake was supported, and indicates that improving either behavior is likely to improve the other. The hypothesis that changes in mood, self-efficacy for controlled eating, and self-regulation for eating would both mediate and be mediated by change in fruit and vegetable intake, with change in exercise as the predictor variable (i.e. reciprocal relationships) was also supported. For mood, this suggests that the previously identified, robust relationship between exercise and mood improvement [51] might minimize emotional or binge eating (which tends to be of high-calorie, high-fat foods [52]), while diet composition may also affect mood [53]. Because maintaining only minimal amounts of exercise has been shown to enhance mood [51], weight-loss treatments could benefit from a strong exercise adherence component focused on supporting manageable volumes. For self-efficacy, the finding suggests that increased exercise fosters feelings of ability to control eating, while improved eating is also associated with an increase in self-efficacy to eat better. When an individual demonstrates to him/herself consistency with exercise – a health behavior considered to be challenging to maintain – this may generalize to feelings of ability for other actions within a family of behaviors commonly known to have health benefits (e.g. improved eating) [21]. As eating improves, confidence in an ability to further extend one’s self in that area may increase. Thus, within treatments, clearly defining even minimal short-term progress toward one’s exercise goals (to increase exercise-related self-efficacy) might lead to confidence in reaching goals related to improved eating. The similar relationships found for increased self-regulation suggest its benefit for improved eating, while improved eating furthers self-regulatory skills usage. As an array of self-regulatory skills (e.g. positive self-talk, self-monitoring) are applied by individuals to overcome numerous barriers to maintaining exercise, progress on better controlled eating may motivate increased usage of such skills directed toward further-improved eating [22,54]. Thus, within treatments, self-regulatory skills that are well-taught in both exercise and eating contexts may provide avenues for their increased development and practice to counter the inevitable challenges to behavioral maintenance or further improvement.

The hypothesis that changes in mood, exercise self-efficacy, and exercise-related self-regulation would both mediate and be mediated by change in exercise volume, emanating from fruit and vegetable intake, was also supported. It was found that changes associated with improved eating that occurred in both mood and exercise-related self-regulation were also both mediators of, and mediated by, change in exercise volume. Progress on eating-related goals is likely to foster better mood and an improved psychological climate for increased exercise [20] while, as discussed before, just the consistent completion of moderate volumes of exercise is likely to promote improved mood. A better understanding of whether specific aspects of the diet improves mood, and/or whether eating-related goal setting (e.g. increase fruit and vegetable intake from 3 servings per day to 5 servings per day within a month) may be manipulated to induce a better mood, is needed. Findings are also consistent with the previous propositions of generalization of self-regulation skills across behavioral contexts [22,54].

Considering the three psychosocial factors accounted for within this research, increased exercise appeared to have a positive association with improvements in *each* eating-related factor tested (self-efficacy, mood, self-regulation), with suggested reciprocality between changes in each psychosocial variable and fruit and vegetable intake; while improved eating demonstrated similar positive associations and suggested reciprocality with the same exercise-related psychosocial factors, excluding exercise self-efficacy. As was indirectly suggested [22,24,54], this supports a behavior-change prediction model positing that changes in self-efficacy, mood, and self-regulation each independently contributes to the explained variance in both improved eating and increased exercise, with a generalization of psychological effects from exercise change to eating change; and may also be interpreted to mean that the overall impact of increased exercise on improved eating may be somewhat more complete than the effect that better eating has on exercise.

Limitations within the present research included the use of change scores that inflated the associated error of the measures used by combining error linked to scores at both baseline and treatment end [55]. Accounting for the dynamic process of changes in exercise and eating behaviors and their psychosocial correlates over the course of treatment was, however, central to the purposes of this investigation. Although the mediation analysis methods applied [28] used a strong resampling procedure to overcome earlier limitations (e.g. a need for normally distributed data), its use within reciprocal effects analyses does not allow multiple variables (that alternated between mediators and dependent variables) to be tested simultaneously. Thus, a total of seven separate reciprocal effects analyses (encompassing 14 mediation analyses) were required which compromised experimental power. Also, there was a somewhat brief 26-week study duration. Because long-term treatment effects related to weight management behaviors has been a great problem, studies over longer time frames that include psychosocial correlates, exercise and eating behaviors, and weight-loss effects are greatly needed [2]. Another limitation was the use of a volunteer sample that might have been highly motivated. Although this is difficult to counter, incorporation of individuals strongly referred by medical professionals might partially address this possible confound. Also, while adequate validation for the present measurement of fruit and vegetable intake was presented, a more comprehensive assessment of the diet (e.g. food diary; full food frequency questionnaire) may benefit extensions of this research (especially where the demands of completing many surveys concurrently are not present, as was the case here). Exercise volume will similarly benefit from more precise measurement methods such as through the use of accellerometry. While social support and expectation effects may bias findings within most field-based research designs, because of the ability to readily generalize findings to applied treatment settings the field nature of this research was considered to be one of its strengths [57,58].

The inclusion of analyses focused on the effects of shorter-term changes (e.g. change in self-efficacy over 3 months) on longer-term outcomes (e.g. change in exercise volume over 1 year) might provide additional insights in extensions of this research. Some researchers, for example, have articulated the advantages of a predictor variable temporally preceding a mediator [56]. Clearly, analytic methods that can better-address temporal aspects and directionality of relationships will improve confidence in findings. Although the selection of psychosocial variables was based on theory and previous research, they did not fully encompass the tenets of social cognitive theory (e.g. effects of social support). Additional psychological constructs, including the omitted aspects of social cognitive theory and those based on other theories applied to health behavior change (e.g. theory of planned behavior), should be tested for their viability.

Because of the substantial contribution that findings within this research might make to behavioral weight-management intervention, considerable replication across sample types and contexts (e.g. cancer survivors, individuals with diabetes, administration within medical centers) is required to ensure confidence prior to adapting treatments accordingly.

## Conclusion

For the present sample of severely obese adults, cognitive-behaviorally based exercise support paired with nutrition support was associated with improvements in volume of exercise, fruit and vegetable intake, mood, and exercise- and eating-related self-efficacy and self-regulation, with greater improvements in exercise, fruit and vegetable consumption, and self-regulation for eating associated with cognitive-behavioral, rather than educational, nutrition methods. There appeared to be a reciprocal relationship between increased exercise and fruit and vegetable intake. Emanating from exercise changes, eating changes suggested reciprocal relationships with improvements in mood and eating-related self-efficacy and self-regulation. Emanating from eating changes, changes in exercise volume suggested reciprocal relationships with improvements in mood and exercise-related self-regulation, but not exercise self-efficacy. After replication, behavioral treatments should focus upon, and leverage, findings relating to the transfer effects from each of these primary weight-loss behaviors to the other in order to improve lagging outcomes.

## Competing interests

The authors declare that they have no competing interests.

## Authors’ contributions

JJA conceived the study design, carried out data analysis, and drafted the report. KJP participated in data analysis, its interpretation, and the writing of the report. Both authors read and approved the final manuscript.
